# TTN novel splice variant in familial dilated cardiomyopathy and splice variants review: a case report

**DOI:** 10.3389/fcvm.2024.1387063

**Published:** 2024-06-13

**Authors:** Paul León, Paula Franco, Nicole Hinojosa, Kevin Torres, Andrés Moreano, Vanessa I. Romero

**Affiliations:** ^1^ College of Biological and Environmental Sciences, Universidad San Francisco de Quito, Quito, Ecuador; ^2^ School of Medicine, Universidad San Francisco de Quito, Quito, Ecuador; ^3^ Department of Cardiology, Universidad de Sao Paulo, Sao Paulo, Brazil

**Keywords:** dilated cardiomyopathy, TTN gene, Ecuador, splicing, truncated variants, case report

## Abstract

This case report details the identification of a novel likely pathogenic splicing variant in the TTN gene, associated with dilated cardiomyopathy (DCM), in a 42-year-old male patient presenting with early-onset heart failure and reduced ejection fraction. DCM is a nonischemic heart condition characterized by left biventricular dilation and systolic dysfunction, with approximately one-third of cases being familial and often linked to genetic mutations. The TTN gene, encoding the largest human protein essential for muscle contraction and sarcomere structure, is implicated in about 25% of DCM cases through mutations, especially truncating variants. Our investigation revealed a previously unreported G > C mutation at the splice acceptor site in intron 356 of TTN, confirmed by Sanger sequencing and not found in population databases, suggesting a novel contribution to the understanding of DCM etiology. The case emphasizes the critical role of the TTN gene in cardiac function and the genetic complexity underlying DCM. A comprehensive literature review highlighted the prevalence and significance of splice variants in the TTN gene, particularly those affecting the titin A-band, which is known for its role in muscle contraction and stability. This variant's identification underscores the importance of genetic screening in patients with DCM, offering insights into the disease's familial transmission and potential therapeutic targets. Our findings contribute to the expanding knowledge of genetic factors in DCM, demonstrating the necessity of integrating genetic diagnostics in cardiovascular medicine. This case supports the growing evidence linking splicing mutations in specific regions of the TTN gene to DCM development and underscores the importance of genetic counseling and testing in managing heart disease.

## Introduction

Dilated cardiomyopathy (DCM—OMIM: 604145) is a nonischemic heart condition characterized by structural and functional abnormalities, primarily marked by left biventricular dilation and systolic dysfunction. Unlike other heart issues, DCM occurs without coronary artery disease, hypertension, valvular, or congenital abnormalities (1). Its etiology encompasses idiopathic factors, exposure to toxins, infections, or metabolic agents; idiopathic cases, lacking identified causes, are presumed to have a genetic contribution. Approximately one-third of cases are familial DCM (FDCM), necessitating genetic counseling, especially when relatives exhibit unexplained heart failure or premature sudden cardiac death (2). In adults, DCM follows an autosomal dominant inheritance pattern, characterized by incomplete penetrance and age-related variable expression (3). Clinically, manifestations vary, ranging from asymptomatic cases to symptoms like dyspnea at rest/exertion, fatigue, abdominal/ankle edema, and angina pectoris. DCM is associated with over 50 genes, with Titin (TTN) gene mutations being the most common, accounting for approximately 25% of cases, particularly truncating variants (4–7). These genetic insights are crucial for understanding the complex landscape of DCM and guiding appropriate management strategies.

The TTN gene encodes a virtual sarcomere component crucial for striated muscle contraction and holds the distinction of being the largest human protein, composed of 364 exons (7–9). TTN's extensive size supports sarcomere structure, aiding contraction (8), facilitating myofibrillogenesis by attaching vital proteins, and mediating cellular processes via its TK domain (9). Seven human TTN isoforms have been identified, comprising approximately a million splice variants theoretically capable of generating from its 364 exons. Among these, cardiomyocytes express three isoforms: N2BA and N2B in adults, and fetal cardiac titin in infants (8, 9).

We report a case involving a family with history of FDCM. Our investigation included panel sequencing, which revealed the presence of a novel likely pathogenic variant within the TTN gene, analyzing its impact will expand our understanding of splicing variants in the TTN gene. We conducted a thorough search in databases to identify splicing variants in the TTN gene that had been previously documented, categorized them based on the specific clinical characteristics they presented.

## Case description

**Figure 1:**
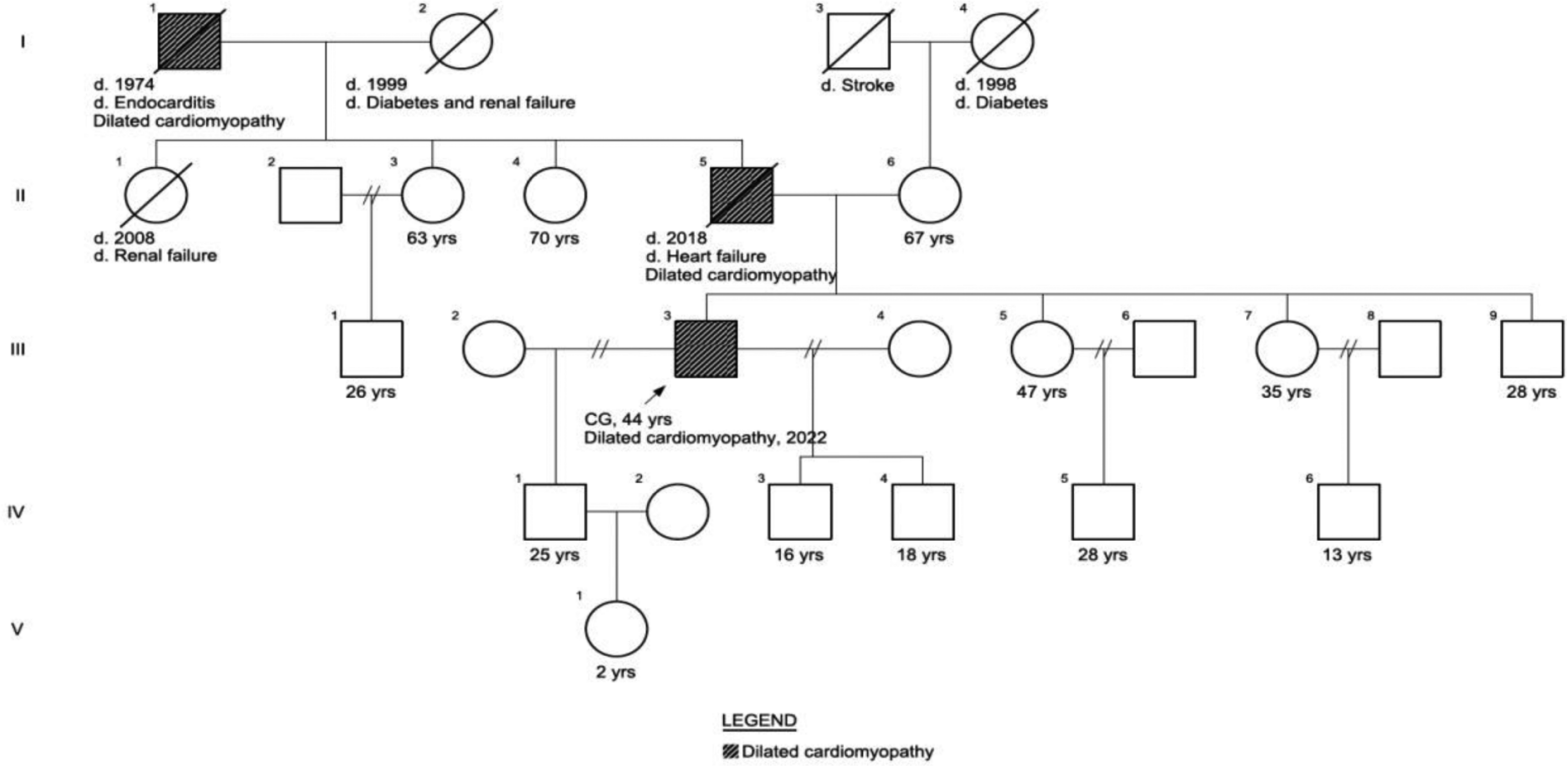
Patient phenotypical pedigree. The patient’s father of the patient died at age 40 due to congestive heart failure and the paternal grandfather died of endocarditis at age 54. The patient has 2 children with different mothers.

A 42-year-old male presented at our genetic outpatient clinic with early-onset heart failure and reduced ejection fraction. He was taking amlodipine, misoprostol, spironolactone, and forxiga. His medical history revealed chest pain during exercise at 18 years old, followed by the onset of dyspnea at 30. By the age of 35, the patient experienced chest pain again, accompanied by severe fluid retention, leading to a hospitalization lasting two weeks. He was treated with diuretics during his hospital stay and was subsequently discharged. However, he discontinued his medication once his condition improved. In June 2022, noticed fluid retention in his feet, a kidney ultrasound was normal and Contrast-Enhanced Urographic Computed Tomography reported prostatic hyperplasia. In November 2022, follow-up laboratory results showed elevated D-dimer (1,818.26 ng/ml), natriuretic peptide (1,843 ng/L), 24 h urine protein (271.25 mg/24 h), aspartate aminotransferase (AST) (57 U/L), total (5.50 mg/dl) direct (3.60 mg/dl) and indirect bilirubin and decreased creatinine clearance (39.18 ml/min). A transthoracic echocardiography revealed a Left Ventricular Ejection Fraction of 24.9%. Remodeling was observed with a tendency toward sphericity, along with a flow gram indicating a restrictive pattern, elevated filling pressures, and reduced systolic function in the right ventricle. The results indicated DCM with severe systolic and diastolic dysfunction of the left ventricle, along with slightly elevated filling pressures and significantly reduced global longitudinal strain. Additionally, the patient exhibited severe biatrial dilatation, mild mitral insufficiency, an intermediate likelihood of pulmonary hypertension, moderate tricuspid insufficiency, and right ventricular systolic dysfunction (Supplementary Figure S1). In December 2022, a 24 h blood pressure monitoring was conducted, revealing nocturnal diastolic arterial hypertension. The father of the patient died at age 40 due to congestive heart failure and the paternal grandfather died of endocarditis at age 54. The patient has 3 children with two different mothers (Figure 1) (Supplementary Figures S1, S2).

## Diagnosis testing

We ordered the Cardiomyopathy Comprehensive Panel that analyzes 121 genes and identified a (heterozygous) G > C c.100172-1 located in the splice acceptor; therefore, this variant affects the splicing process and consequently the damages the protein (Supplementary Figure S3). It is categorized as a likely pathogenic variant in intron 356 of TTN. This variant has not been reported in the literature in individuals affected with TTN-related conditions and is not present in population databases (gnomAD no frequency). The splice variant was confirmed by Sanger sequencing. We carried out Sanger sequencing on six family members and identified the variant in the patient's older sister and middle son. However, neither of them exhibited cardiac symptoms. We suggest that the variant exhibits no penetrance in the sister and recommend a cardiac evaluation for the middle son.

Our patient variant was reported as likely pathogenic; however, our findings suggest a pathogenic effect. The variant is not present in population databases (PS4 PM2 BA1 BS1 BS2), the domain is known to be critical to the protein function (PM1), there is evidence of pathogenicity in other studies (PM4 BP3), the patient has a well-defined clinical characterization including a possible consistent mode of inheritance- dominant (PP4).

## Review of splicing variants

**Figure 2:**
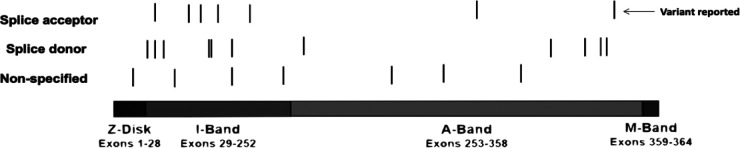
Graphical description of the TTN variants. We identified 35 reported cases of TTN variants in the splicing regions (Table 1) and constructed a visual representation of the reported variants along TTN. Splicing variants are the lines below the image, the black line means there was a variation reported in that specific location. TTN bands are shown—Z-disc, I-band, A-band, M-band. There are 1 variant located in the Z-disk, 14 variants in the I-band and 11 variants in the A-band. The arrow indicates our reported variant. Furthermore, for 2 variants reported the location its unknown and therefore, are not included in the graph.

We conducted a comprehensive literature review including reported cases of splice variants in TTN, focusing on adults with early-onset heart failure. After filtering, we identified 35 documented cases. Our findings align with what Patel et al. (10) suggested when they mentioned that mutations occurring in nucleotides flanking either the 5′ exon's end (donor site) or the 3′ exon's start (acceptor site) are characterized as likely pathogenic or pathogenic with mutations on the 3′ acceptor site being more prevalent. The splice donor site consists of nine base pairs that encode the invariant dinucleotide (GT) found at the 5′ end of nearly all introns. The splice acceptor site, spanning 23 base pairs, includes an invariant dinucleotide (AG). Mutations impacting the GT or AG dinucleotides within highly expressed exons are identified as likely pathogenic or pathogenic, leading to truncation variants of the TTN gene. Mutations occurring within the remaining seven base pairs of the donor site, or the 21 base pairs of the acceptor site may potentially disrupt splicing and are classified as variants of unknown significance (10). A significant proportion of pathogenic splice variants were identified within exons or introns located in the titin A-band (Figure 2). This region is characterized by a high frequency of constitutively expressed exons and consists of alternating fibronectin and immunoglobulin (Ig) regions, which serve as anchors for myosin binding during muscle contraction (8). However, most mutations that lead to DCM are found within the fibronectin type III domain of the A-band (11).

**Table 1:** TTN splice variants of 35 reported cases.

Sex	Age (years)	Band	Type of mutation	Splice site (bp)	Disease	References
NR	NR	Z-disk	Non-specified	p.32991_32992del	DCM	Akinrinade et al., (11)
M	15	A-band	Splice donor	c.98671–2 A > G	Myopathy, early-onset, with fatal cardiomyopathy	Savarese et al. (12)
NR	NR	A-band	Splice donor	c.97492 + 1G > A	Peripartum cardiomyopathy	LaDuca et al. (13)
NR	NR	I-band	Splice acceptor	g.179407388delC	Left ventricular noncompaction	Miszalski-Jamka, K. et al. (14)
NR	NR	A-band	Splice donor	c.95416 + 1_95416 + 2insCC	Cardiomyopathy, dilated	Roberts et al. (15)
NR	NR	A-band	Splice donor	c.86821 + 2T > A	Cardiomyopathy	Wu et al. (16)
NR	NR	A-band	Non-specified	c.59933-1 G > C	DCM	Akinrinade et al., (11)
NR	NR	A-band	Non-specified	c.67637-1G > C.	DCM	Patel et al. (10)
NR	NR	I-band	Non-specified	c.63881 + 5G > A	Cardiomyopathy	Hazebroek et al. (17)
F	NR	A-band	Splice donor	c.51436 + 1G > A	DCM	Roberts et al. (15)
NR	NR	A-band	Splice acceptor	c.74837-74840dupTTAG	Congenital myopathy	Felkin, et al. (18)
F	NR	A-band	Non-specified	NR	DCM	Patel et al. (10)
NR	11	I-band	Splice acceptor	c.44816-1G > A	Centronuclear myopathy	Ceyhan-Birsoy et al. (19)
NR	NR	I-band	Non-specified	TCTTCT TGCCCT CCACC TCAG/p.32991_32992del	Congenital myopathy	Ceyhan-Birsoy et al. (19)
NR	NR	I-band	Splice donor	c.31484-286G > T	Cardiomyopathy	Gaertner et al. (20)
NR	NR	I-band	Splice donor	c.31484 + 1715A > C	Cardiomyopathy	Ware et al. (21)
NR	NR	I-band	Splice donor	c.34930 + 2T > C	DCM	
M	2	A-band	Non-specified	NR	DCM	Patel et al. (10)
NR	NR	I-band	Splice acceptor	c.28114 + 1 G > A	Congenital myopathy	Ceyhan-Birsoy et al. (19)
NR	NR	I-band	Splice acceptor	c.31763-1G > A	Cardiomyopathy	Meng et al. (22)
F	13	I-band	Splice donor	c.10361-1522 G > A	Cardiomyopathy; Myopathy; Muscular dystrophy	Schafer et al. (23)
NR	5	I-band	Splice donor	c.15721 + 1G > A	Myopathy	Tian et al. (24)
NR	NR	I-band	Non-specified	g.179616771delAA	Hypertrophic cardiomyopathy	Schafer et al. (23)
NR	NR	I-band	Splice donor	c.11254 + 2T > C	Cardiomyopathy	Gaertner et al. (20)
F	0,6	I-band	Splice acceptor	c.24730 + 1 G > T	Myopathy, early-onset, with fatal cardiomyopathy	Bryen, S. J., et al. (25)
M	27	A-band	Non-specified	c.921611 + 3A > T	limb-girdlemusculardystrophytype2G	Oates et al. (26)
F	41	M-band	Splice acceptor	c.107780_107790delinsTGAAAGAAAAA	Mild DCM	Hoorntje et al. (27)
M	47	M-band	Splice acceptor	c.107889del	DCM	Klauke et al. (28)
NR	NR	A-band	Splice acceptor	c.63794-1G > A	DCM	Deciphering Developmental Disorders Study, (29)
M	49	A-band	Splice donor	c.59926 + 1G > A	DCM	Ware et al. (21)
NR	NR	A-band	Splice acceptor	c.100766-1G > T	DCM	Zenagui et al. (9)
NR	NR	A-band	Splice donor	c.51436 + 1G > A	DCM	Gonorazky, H. D., et al. (30)
M	31	A-band	Splice acceptor	c.49346-1G > A	DCM	Trujillano et al. (31)
NR	NR	I-band	Splice donor	c.16621 + 1G > T	DCM and Mild peripheral pulmonary stensosis	Hoorntje et al. (27)
M	43	I-band	Splice acceptor	c.14372-2A > G	DCM	Deciphering Developmental Disorders Study, (29)

NR, not reported; DCM, dilated cardiomyopathy; F, female; M, Male; c., coding.

## Discussion

We present a case of a 42-year-old male patient with severe fluid retention, prostatic hyperplasia, and diastolic arterial hypertension. Genetic sequencing of this patient revealed a novel splice variant located in the A-band intron 356. Akinrinade et al. (11) compiled data from 1,000 Genomes Project, Exome Sequencing Project (ESP) and Exome Aggregation Consortium (ExAC) and found that TTN variants affecting splice sites are linked to early muscle weakness, reduced reflexes, and respiratory issues. These symptoms are similar to the ones exhibited by our patient. This occurs because these types of mutations disrupt exon acceptor sites, leaving transcripts unspliced and leading to truncated titin protein (11). Patel et al. (10) noted that individuals DCM frequently possess TTN variants located a few base pairs away from the donor or acceptor splice sites. In our study, the identified mutation is situated just one base pair from the acceptor splice site of a constitutively expressed exon (Table 1). In fact, exon 356 has a PSI score of 100% meaning that the proportion of TTN transcripts that incorporate this exon based is 100% in DCM, Even though some residues contribute to intron splice guiding, the nucleotide flanking either the 5′ exon at the donor sire or the start of the 3′ exon on the acceptor site are the most comonly mutated in patients with DCM (7). The acceptor site spans for about 23bp at the 3′ exon boundary which encapsulates the AG splice acceptor site. Multiple studies have reported that variants affecting the AG region of the acceptor site in exons with high PSI scores are often categorized as pathogenic TTN variants (2, 7, 15) Huang et al. (32) reported a similar case of DCM with a mutation in the A-band splice variant in exon 343 affecting all isoforms. Most of the TTN truncation variants including splicing leading to DCM exhibit a position-dependent impact with more severe symptoms with mutations closer to the C-terminus (33). Furthermore, TTN variants located in the I-band are more tolerated since many exons on this area are subject to alternative splicing (7, 33). Herman et al. (34) also noticed the absence of DCM-associated mutations in the Z and M-band subunits. Our case emphasizes the significance of the novel A-band splice variant underlining its role in DCM development and progression.

The TTN protein is the largest one in humans and is divided into four functional regions. These are the N-terminal Z-line which anchors to the sarcomeric Z-disk. Whereas the I-band is responsible for providing the protein's elastic properties since it consists of tandem Ig segments of linked Ig-like domains (7). The A-band is inextensible and binds myosin and myosin-binding protein C (MyBP-C) to provide a stabilizer role of the thick filament. Finally, the M-line, embedded in the C-terminal, expresses a kinase that regulate gene expression and cardiac remodeling (7).

DCM is a primary myocardial condition caused by a combination of genetic and non-genetic components with a variable clinical presentation. However, even the non-genetic types of DCM can potentially be influenced by the patient's genetic profile such as suffering from hypertension, valve disease, and inflammatory responses (35). Around 30% of DCM cases have been attributed to a familial genetic origin where mutations in the TTN protein have been shown to be the major cause for DCM (7). The TTN protein has a major role in cell structure, muscle elasticity and interacts with multiple protein partners which means that different mutations could have a significantly different impact leading to diverse clinical manifestations depending on each case (36). For instance, Roberts et al. (15) found that patients having TTN truncation mutations show lower stroke volume, thinner left ventricle walls and more severe impaired left ventricle function in comparison with non-familial cases of DCM. Moreover, patients with TTN truncations variants tend to suffer from sustained ventricular tachycardia (7) but exhibit a good response to medical treatment (37). Furthermore, in a multisite study of patients with DCM, Hofmeyer et al. (38) noted that patients with advanced DCM, meaning that they need a left ventricular assist device or a heart transplant, were twice as likely to carry a TTN truncation variant compared with non-advanced DCM patients. Consequently, the location where the mutation takes place in the TTN protein will determine the biological impact with variants located in the A-band being more likely to result in worst cases of DCM (7).

A limitation for this study was that, even though some databases such as the HGMD and PubMed show a considerable number of TTN splicing variants reported, once we look at every variation mentioned in papers, most of them are either missense or nonsense. This became an issue since the number of true TTN splicing variants reported is lower and we have less cases to compare with ours.

## Conclusion

TTN, the largest human protein with significant heart expression, plays a role in elasticity, passive force during diastolic and systolic functions, and sarcomere assembly/length (36). Here, we describe the case of a patient with DCM harboring an intron 356 splice mutation in the TTN gene as the likely cause for his condition, accompanied by a literature review of splicing variants from PubMed and HGMD. Our results further support evidence that links splicing mutations in the A-band encoding region with the development of DCM in comparison with mutations in other regions of the TTN gene.

## Data Availability

The original contributions presented in the study are included in the article/Supplementary Material, further inquiries can be directed to the corresponding author.
