# Supplementary material for: TTN novel splice variant in familial dilated cardiomyopathy and splice variants review: a case report
Source: Front Cardiovasc Med. 2024 Jun 13;11:1387063. doi: 10.3389/fcvm.2024.1387063 (PMC11210389; doi:10.3389/fcvm.2024.1387063)
Supplement: Supplementary file 1 [file Datasheet1.docx]

Supplementary Material

# Supplementary Data

**Supplementary figure 1.** Heart rate= 99bpm (beats per minute), LEFT VENTRICLE Simpson catheter, VFD(Venous flow velocity) =146.72ml, EDV Index(End-diastolic volume ) = 78.29, SV(Stroke volume) = 49.57ml, CO(Cardiac output) = 4.858 l/min, EF(Ejection fraction) = 33.79%, VFS(A4C) = 88.61ml, ESV index(End systolic volume) = 47.28, SI(Systolic index) = 15.7, CI(Cardiac index) = 1.54, VFS(A2C) = 97.15ml, ESV Index(End systolic volume) = 51.84, SI(Systolic index) = 26.45, CI(Cardiac index) = 2.59, VFD(Ventricular Flow d) =118.03ml, EDV Index(End distolic volume) = 62.98, SV(stroke volume) = 29.42ml, CO(Cardiac output) = 2.883 l/min, EF(Ejeccion Fraction) = 24.92%, LEFT ATRICLE AI Vol() = 134.04m, LA Vol Index (Left atrium volume index) = 71.52, AI Vol(Aortic insufficiency volumen) = 134.04ml, LA Vol Index (Left atrium volume index) = 71.52, RIGTH ATRICLE AD Vol() = 120.61ml, RA Vol Index (Right atrium volume index) = 64.36, AORTA AND AORTIC VALVE AI/AO (2D) Diameter AI = 46.3mm AI/AO (atrium/aorta)= 1.52, Aorta diameter = 30.6 mm, MEASUREMENTS LEFT VENTRICLE SIVd = 8.5mm, SIVs (septum internal volume)= 14.2mm, DIVId (DI Internal end diastolic diameter)= 57.7mm, DIVIs (DI Internal end systolic diameter) = 46.3mm, PPVId (PP internal end diastolic diameter)= 7.3mm, PPVIs (PP internal end systolic diameter)= 8.9mm, RWM (Regional wall motion)= 0.25, SIVd/PPVId = 1.17, SIVs/PPVIs = 1.59, VI Mass(VENTICULAR INDEX MASS) = 200g, VI Mass-I(VENTRICULAR INDEX MASS -I) = 106.90, TEICHHOLZ VFD(Teich) = 164.57ml VFS (Teich) = 98.93ml, SV(store volume Teich) = 65.64ml, FE(Ejection Fracción Teich) = 39.89%, EDV Index (End diastolic volume index Teich) = 87.82, ESV index(End systolic volume index Teich) = 52.79, SI(Septum internal Teich) = 35.03, TRICUSPID VALVE TAPSE (Tricuspid Annular Plane Systolic Excursion) = 14.9mm, MITRAL VALVE E ´(Peak velocity of early diastolic mitral annular motion as determined by pulsed wave Doppler) VELOCITY = 91.04cm/s, MV E PG (MITRAL VALVE PEAK PREASURE GRADIENT)= 3.39mmHg, VM A Vel (Volume mitral velocity)= 38.23cm/s, MV A PG (MITRAL VALVE PRESSUARE GRADIENT)= 0.58 mmHg, E/A VM (relationship between early ventricular filling wave and atrial contraction) = 2.41, Tdec VM (Time decesaleration Mitral valve) = 133ms, AORTIC VALVE Vmax (velocity max) = 69.68cm/s, PGmax VA (Pressure gradient max aortic valve) = 1,94mmHg, TRICUSPID VALVE Vmax = 274.38cm/s, TV PGmax (Tricuspid valve pressure valve) = 30.11 mmHg, PULMONAR VALVE VP AccT (acceleration time)= 95ms, TDI (Tissue doppler imaging) measurements VM media Vel.Septal E’VM = 3.77cm/s,MV E/E’ Septal (mitral valve annular velocity (E/E') septal)= 24.38, VM lateral Vel lateral E’VM (lateral mitral annular velocity) = 6.48cm/s, MV E/E’ lateral (mitral valve annual velocity lateral)= 14.19, MV E/E’ (mitral valve velocity peak)= 17.94


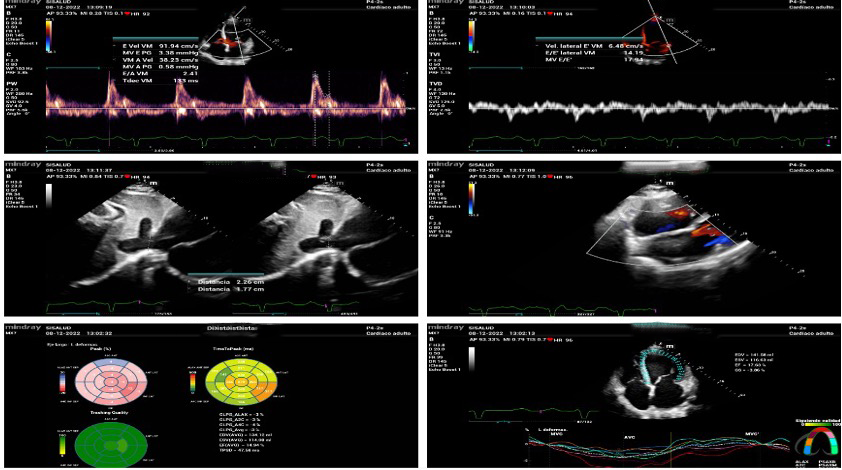


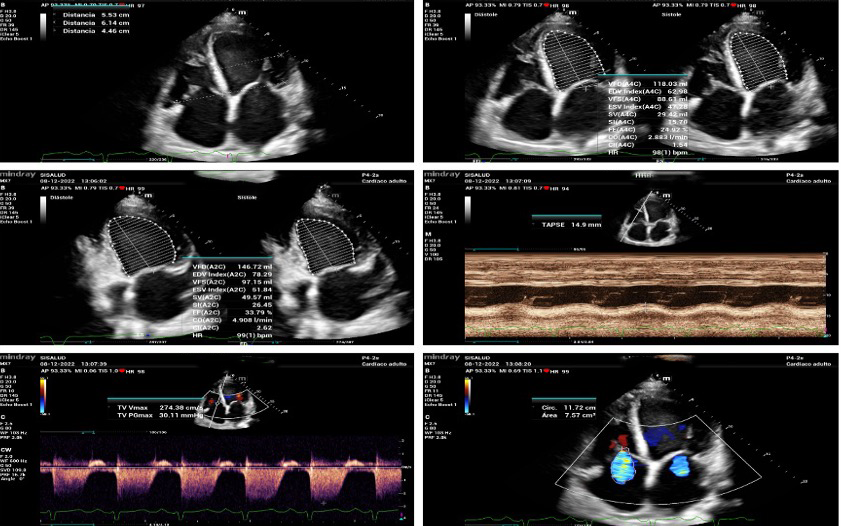


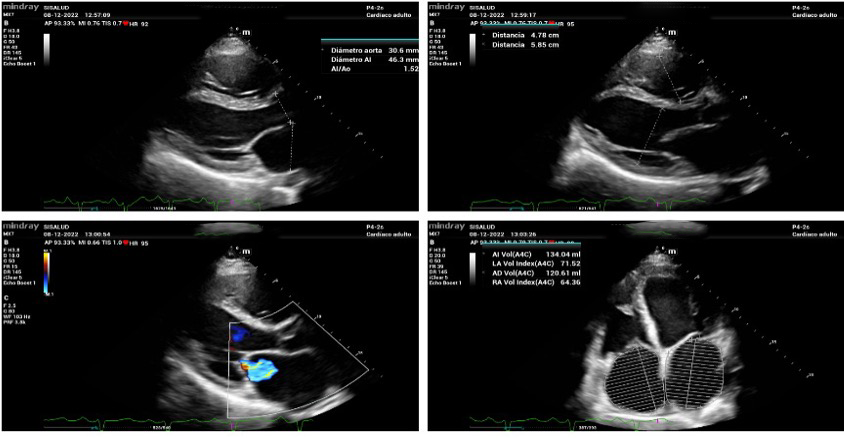


**Supplementary figure 2. CARE timeline**
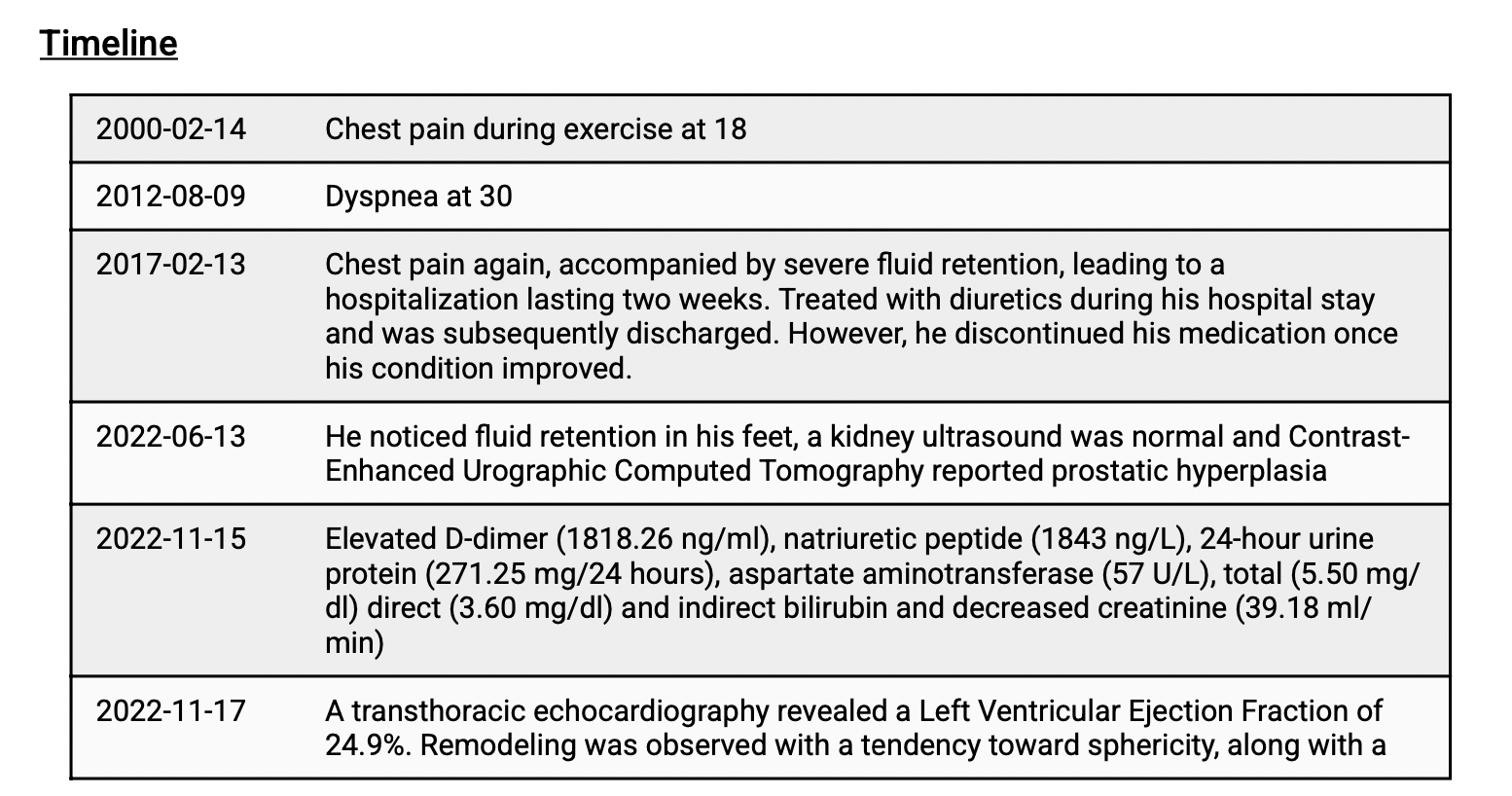


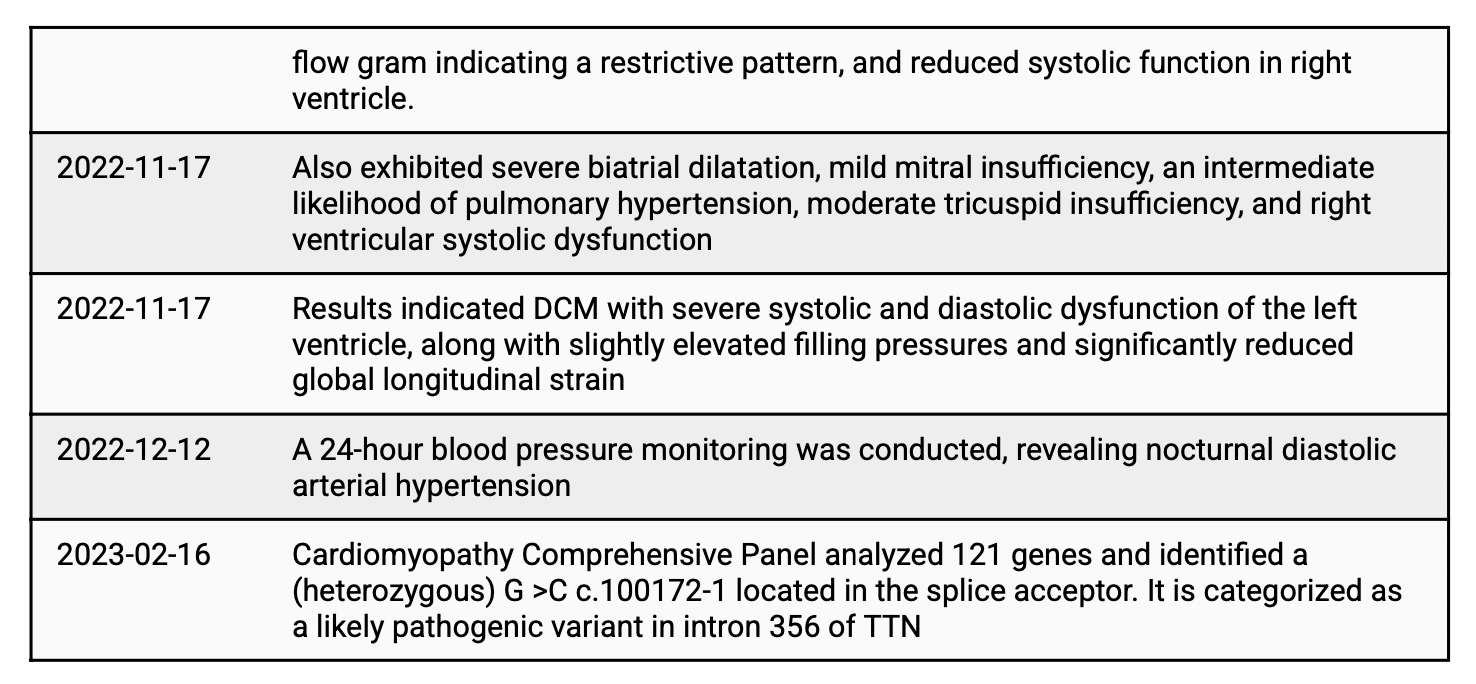


Supplementary table 1. Genes analyzed in the Cardiomyopathy Comprehensive Panel

| **Genes analyzed in the Cardiomyopathy Comprehensive Panel** |
| --- |
| - A2ML1 - ABCC9 - ACADVL - ACTC1 - ACTN2 - AGL - ALMS1 - ALPK3 - ANKRD1 - BAG3 - BRAF - CACNA1C - CALR3 - CAV3 - CBL - CDH2 - CHRM2 - CPT2 - CRYAB - CSRP3 - CTF1 - CTNNA3 - DES - DMD - DNAJC19 - DOLK - DSC2 - DSG2 - DSP - DTNA - ELAC2 - EMD - EYA4 - FHL1 - FHL2 - FKRP - FKTN - FLNC - GAA - GATA4 - GATA6 - GATAD1 - GLA - HAND1 - HCN4 - HRAS - ILK - JPH2 - JUP - KIF20A - KLF10 - KRAS - LAMA4 - LAMP2 - LDB3 - LMNA - LRRC10 - LZTR1 - MAP2K1 - MAP2K2 - MAP3K8 - MED12 - MRAS - MTO1 - MYBPC3 - MYH6 - MYH7 - MYL2 - MYL3 - MYLK2 - MYLK3 - MYOM1 - MYOZ2 - MYPN - NEBL - NEXN - NF1 - NKX2-5 - NPPA - NRAS - PCCA - PCCB - PDLIM3 - PKP2 - PLEKHM2 - PLN - PPCS - PPP1CB - PRDM16 - PRKAG2 - PTPN11 - RAF1 - RASA1 - RASA2 - RBM20 - RIT1 - RRAS - RYR2 - SCN5A - SDHA - SGCD - SHOC2 - SLC22A5 - SOS1 - SOS2 - SPRED1 - TAZ - TBX20 - TCAP - TMEM43 - TMEM70 - TMPO - TNNC1 - TNNI3 - TNNI3K - TNNT2 - TPM1 - TTN - TTR - TXNRD2 - VCL |
